# A Comprehensive Review of Artificial Intelligence Methods in Bone Age Assessment

**DOI:** 10.1049/htl2.70056

**Published:** 2026-02-11

**Authors:** Mohsen Borjalizadeh, Farshid Babapour Mofrad, Midya Yousefzamani

**Affiliations:** ^1^ Department of Medical Radiation Engineering SR.C., Islamic Azad University Tehran Iran

**Keywords:** bone, diagnostic radiography, learning (artificial intelligence)

## Abstract

Bone age reflects individual skeletal maturity and is an important factor in the follow‐up and monitoring of growth and development in children. Determination of bone age by paediatricians has remained one of the most typical indications that require the use of radiology, and the historical method by which radiologists determine bone age is from a bone age atlas. However, there are still some challenges. The limited case diversity related to race, geography, and age distribution; small sample sizes; and the lack of expert validation by multiple radiologists limit the generalisability of current models. Many underlying comorbidities or health histories are often overlooked in developed models. The models of the future, which provide greater accuracy and clinical usefulness, must encompass more diverse datasets, more thorough health histories, expert validation, and fast but reliable artificial intelligence (AI) models. As an educational review, this study analysed a variety of AI‐based approaches that have emerged in the past several years for paediatric bone age assessment (most using hand and wrist radiographs and often coupled with radiology reports). Of these, models such as RCNN, which we evaluated with mean absolute error, showed great potential. There are great future clinical applications and advancements that can progressively transform bone age assessment and evaluation from AI. Notably, we did identify the gaps and opportunities for potentially improving the future clinical approach of bone age assessment and evaluation.

## Graphical abstract

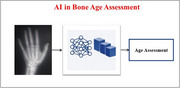

## 1 Introduction

Determining bone age (BA) is very important for evaluating glandular diseases in children as well as the rate of biological growth of the body and comparing it with chronological age [1]. This test is usually prescribed by paediatricians or paediatric endocrinologists. BA assessment (BAA) helps to monitor and evaluate the speed or slowness of a child's growth and development over time. This helps specialists to identify developmental disorders or abnormalities [2]. Paediatricians and orthopaedic specialists use BA to determine the most appropriate treatment for conditions such as developmental delay or early puberty [3]. By assessing BA, medical professionals can predict how much longer a child will grow, when a child will begin puberty, and what will be the final height of the child, which is especially useful for children who are prone to short stature [4]. The test can also help doctors monitor progress and guide treatment for children with conditions that affect their growth, including diseases that affect growth hormone levels, such as hypothyroidism, precocious puberty, and adrenal growth hormone deficiency gland disorders, and genetic developmental disorders, such as Turner syndrome and orthopaedic or orthodontic problems, where the time and type of treatment are guided by the expected growth of the child [5]. Artificial intelligence (AI) algorithms can help with these diagnostic processes by integrating clinical data and radiographs to identify abnormal patterns in bone growth that can be due to endocrine or genetic disorders in order to make the intervention more individualised and timely [6].

Basically, there are different methods to evaluate BA. These methods include radiography, ultrasound, MRI, etc. [7, 8]. Considering that radiography is affordable and available, it is the most used in BAA. Radiographs of the neck, wrist, elbow, knee, and teeth can be used in BAA [9, 10, 11, 12]. Among them, radiography of the wrist and hand is more useful. Most radiologists, due to the existence of abundant data, as well as the atlas of the wrist and hand region, prefer to use this area to evaluate and estimate BA [13]. Determination of BA by wrist and hand radiographs is reported by radiologists in two ways. The first method is through the Greulich–Pyle (GP) atlas [14]. This atlas includes a complete set of wrist and hand radiographs in the range of 1 month to 18 years for girls and boys. Radiologists compare the patient’s wrist and hand radiograph with the atlas images to estimate and report the patient’s bone age (BA). The second method is Tanner‐Whitehouse (TW) [15], which divides the wrist and hand into different areas, and a score is assigned to each area based on age and race, and as a result of the sum of all these scores, the BA is determined. This method is more accurate than GP, but it takes more time. As a result, experts prefer to use the GP method [16]. Although GP is still more commonly used because it is easier to understand and use, TW is considered to be more accurate in that it employs a region‐wise scoring method. Other emerging measures, such as MRI‐, ultrasound‐, and dental‐based methods, have also been examined in certain circumstances and populations [13]. Manual interpretation using GP or TW is not only time‐consuming, but it is also susceptible to inter‐observer variability. AI‐based models – like CNNs – are able to analyse radiographs within seconds and consistently reduce the amount of time it takes to come to a diagnosis, while also limiting human error. For example, fully automated CNN systems have demonstrated similar or even superior performance to experienced radiologists when estimating BA within large datasets [17]. Other methods to assess skeletal maturity have been developed to ease accuracy and applicability across age groups from GP and TW. The GP atlas, mentioned earlier, provides high‐resolution digital reference points, although variability related to outlier (atypical) points remains a limitation [18]. The Sauvegrain (SG) method evaluates elbow ossification centers and is particularly precise during puberty. In contrast, the GP method has limitations during rapid adolescent maturation, especially between 11 and 15 years of age [19]. There is generous variability in the speed, accuracy, and applicability of the methods across populations. TW will likely produce more useful data compared to the other methods, although it requires more time and expertise than the methods tested. The manual approaches we presented offer a comparative platform considering how AI‐based systems could support or even enhance the work of GP/TW systems [20]. In recent years, with the expansion of AI applications in image processing, BAA by AI algorithms has increased [17]. AI includes a wide range of research, and various problems can be solved using AI models and methods. However, AI research areas can be divided into several sections, branches, or trends. The field of machine learning (ML) is considered as one of the most important and main trends in AI, and many researchers and activists in the field of AI use ML algorithms to develop intelligent software and systems. By using ML methods, it is possible to build intelligent systems that automatically learn how to solve different problems. The learning of ML‐based systems is based on the available data and the statistical identification of their patterns, and with the analysis of more data, the accuracy of their performance increases. With the emergence of the field of deep learning (DL), a concept called convolutional neural network (CNN) also emerged, which can implement different types of DL methods using the CNN architecture. The CNN consists of several layers, the first and last layers of which are responsible for receiving input and providing output, respectively. The middle layers of the network also identify patterns in the input data so that the output of the model can be determined based on them [21, 22, 23]. In recent years, CNN has been used for the analysis of medical images in particular, including the following: tumour detection [24], detection and classification of micro‐calcifications [25], and classification of X‐ray images such as chest and BA [26]. Since determining BA with the help of AI can take less time than manual methods, artificial neural networks (ANNs) as a sub‐branch of AI and DL have become one of the goals of researchers. Therefore, researchers are trying to provide algorithms and design better architectures, the best results with the least errors [6, 27, 28, 29, 30]. While we have seen some advances, there is still a significant research gap in the literature. Different studies have relied on limited datasets with little variation in age, race, and geographical background. Further, only a handful of studies used multiple expert validations, and few considered any underlying health conditions impacting growth patterns for BA determination. The research project aims to address the gaps outlined and provide a systematic evaluation of AI‐based approaches for BA assessment while identifying limitations and challenges in BA assessment today. Given the increasing use of AI algorithms in various fields of medical imaging, and especially BAA, there is a need for a coherent and educational review to identify the current state of research in this field. Although the studies published so far have provided valuable results, the dispersion of methods and diversity of approaches have made it difficult to achieve a comprehensive view. Therefore, the aim of this study is to conduct an education review of the existing evidence on AI methods used in BAA and summarise the findings, research trends, existing challenges, and future research gaps.

This article begins with a comprehensive methods section detailing the motivation, research questions, article selection strategy, and dataset characteristics. It then reviews important trends in AI‐based BAA in terms of imaging modalities, model types, and evaluation metrics. By synthesising findings from over a decade of research, significant gaps in this research area are reported, including an insufficient degree of diversity among populations studied, the lack of multi‐modal or multi‐expert validation, and a need for longitudinal datasets. The discussion synthesises the findings and underscores the relatively high predictive performance of deep‐learning models, including RCNN and VGG, especially when evaluated in terms of mean absolute error (MAE). Ethical considerations and clinical applicability are outlined. Finally, the article concludes with future directions such as the development of population‐specific models, the use of transformer‐based methods, and the importance of explainability in the adoption of AI in clinical practice.

## 2 Literature Search Method

**FIGURE 1:**
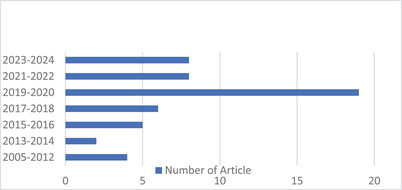
Distribution of extracted articles across different periods.

The articles collected in this review have been prepared from reputable journals. The data in this study regarding the intelligent determination of artificial BA were collected with the help of Google Scholar, Scopus, PubMed, and ScienceDirect, with an effort to extract reliable sources. In this study, numerous published articles on BAA using AI from a broad time span were reviewed (Figure 1 shows the frequency of extracted articles by year). The selected period focuses on the most recent ten years, which represent the most important developments in the field of AI, particularly in DL. AI applications have started to have an impact on clinical practice, and this represents the time when AI in medical imaging has matured from experimental work to practical solutions. The differences among these articles lie in the implementation method, the tested area, the type of modality, the results obtained, and the type of evaluation. While reviewing the selected literature, it was noted that different studies used different evaluation measures depending on the type of task. For those BAA models based on regression, accuracy was measured in terms of the MAE and the root mean square error, indicating the numerical accuracy of the prediction. In those works using a classification approach, accuracy, precision, recall, F‐score, and Matthews correlation coefficient were generally used as measures of the diagnostic accuracy of the models. The variation in evaluation methods reflects differences in data distributions, learning objectives, and validation processes employed in the reviewed studies.

In this study, an overview of all the mentioned cases has been done. In the selection of articles, the following points were considered: In which journal was the article published? The date of publication of the article, the number of references to the article, and the quality of the article. Furthermore, priority was given to articles that displayed methodological rigour; for example, being explicit about the research design, including a conceptual or theoretical framework, describing the statistical analysis, specifying the population and the sampling strategy, and articulating the relationships between the important variables.

Figure 1 shows the annual variation in publication output in the area of BAA with AI. The data indicated a significant growth in publications during 2019–2020, with publications peaking to a level we have not seen in this field. This growth may indicate increased academic and clinical interest in using AI for BAA. Moderate publication levels are noted for 2023–2024 and 2021–2022, indicating recent contributions, while lower frequencies are evident for 2017–2018 and 2015–2016, suggesting a gradual rise in research output. No activity is recorded during the pioneer period or the 2013–2014 interval, probably because the field was still in its nascent stage. This distribution shows the ever‐evolving research trends driven by technological progress and focus shifts within the field. The review primarily includes original research articles, systematic reviews, experimental studies, and select clinical trials, with a focus on peer‐reviewed journal publications in the fields of radiology, paediatrics, and biomedical engineering. The purpose of this study is the general review of BAA methods and their challenges. Also, in this article, an attempt has been made to compare and discuss the results of BA estimation methods based on AI. The main goal of this article is to find out which method and model show the best results and the lowest errors. Three questions are asked as a subset of the main question:

**RQ1** Can intelligent BA estimation methods be used for diagnostic aid for radiologists and endocrinologists?

**RQ2** How reliable can AI algorithms be for endocrinologists and radiologists?

**RQ3** What are the effective factors in accurate estimation of BA based on AI?

**FIGURE 2:**
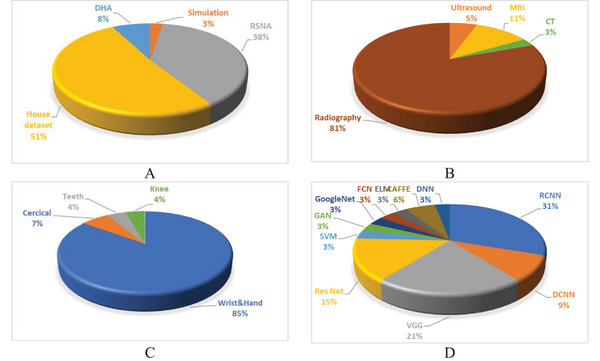
Analysis of data sources, imaging techniques, targeted organs, and AI models in bone age assessment. (A) Frequency percentage of datasets in this study. According to the data of the articles, the datasets have been more than the House data. Also, RSNA is one of the popular sources for researchers. (B) Frequency of imaging modalities to assess BA in per cent. There are different modalities to assess BA, among which radiography is more useful than others. Radiography has a lower cost than other modalities and is also available. Therefore, it is the most used in BA estimation. (C) Frequency of organs in percent. BAA may be done from different organs of the body, but radiography of the wrist and hand is more common. The wrist and hand can be further evaluated for various reasons. One of these cases is the presence of GP and TW atlases. (D) Percent frequency of AI‐based models obtained from extracted articles.

Figure 2 provides a general overview of important phases in BAA relating to the use of datasets, imaging techniques, anatomical sites, and AI‐based models. The first figure shows the distribution of datasets, considering the House and Radiological Society of North America (RSNA) datasets as the most frequently used datasets in BAA. The second figure shows the different frequencies of used imaging techniques in BAA, where it can be established that radiography is the prime method used due to cost‐effectiveness and its general accessibility. The third figure concentrates on anatomical sites of BAA, which frequently include the wrist and hand as the primary sites owing to substantial association with skeletal maturity and also the availability of standard reference atlases. The fourth figure focuses on the distribution of AI‐based models, where CNN‐based architectures such as RCNN, VGG, and ResNet are extensively used for automated BA estimation. Thus, these figures present a summary of the different methodologies and advances made in this direction within BAA research.

### 2.1 Data Description

Numerous articles on BA evaluation and estimation methods with the help of AI have been extracted from a broad time span. The important characteristics of these articles can be described as follows: the number of tested data, gender and race, the number of experts who reported findings, the tested area, the source of data extraction, the age range of the data, the tested age intervals, the type and method of DL, and the type of modality used for imaging.

#### 2.1.1 Search Strategies

In this educational review, an exhaustive search was performed through reputable electronic databases (e.g., PubMed, Scopus, and IEEE Xplore). Articles limited to the English language published between 2005 and 2024 with the full text available that directly addressed the subject of the application of AI to BAA were considered eligible. In order to broaden the comprehensiveness of this search, the references of the articles which were identified were read so that further articles which pertained to the subject could be ascertained. Inclusion criteria were restricted to original articles. Exclusion criteria consisted of review articles, case reports, letters to the editor, articles not permitting access to their full texts, papers in languages other than English, articles which were not published within this timeframe, and papers that were recognised not to pertain to the subject presented. From the very first count of 108 articles recognised, 15 were removed as duplicates. Of these 93, 11 were then eliminated as articles which would not permit access to their full texts and 32 as articles irrelevant to the subject. Ultimately, of the remaining articles, 50 fulfilled the inclusion criteria and were consequently embraced for qualitative analysis. No attempt was made to recoup articles recognised but unpublished. No attempt was made to retrieve unpublished articles or data, except where such material was referenced in the identified studies, to minimize publication bias. The included articles were critically examined by means of the QUADAS‐2 tool in order to estimate the risk of bias and applicability. A second investigation was conducted in which papers assigned a high risk of bias were excluded, and the results are presented in Table 1. The results of this investigation are considered in relating the relevancy of the investigation that had been conducted.

**TABLE 1:** Summary of the frequency distribution of risk of bias and applicability concerns across QUADAS 2 domains.

Domain	Low risk (count, %)	Moderate risk (count, %)	High risk (count, %)
Patient selection bias	33 (66.0%)	17 (34.0%)	0 (0%)
Patient selection applicability	7 (14%)	35 (70%)	8 (16%)
Index test bias	43 (86.0%)	7 (14.0%)	0 (0%)
Index test applicability	7 (14%)	35 (70%)	8 (16%)
Reference standard bias	44 (88.0%)	6 (12.0%)	0 (0%)
Reference standard applicability	6 (12%)	34 (68%)	10 (20%)
Flow and timing bias	20 (40%)	30 (60%)	0 (0%)

#### 2.1.2 Modality

Various modalities such as radiography, MRI [31, 32, 33, 34], ultrasound [35], and CT scan [36] are used to determine BA. Meanwhile, radiography is used more than other methods due to the following reasons: affordability, availability, and the presence of different atlases for BA interpretation. Also, preparation of BA radiology can usually be done in a few seconds. For X‐rays of the child's bones, it is enough to expose the child's left hand to a standard amount of X‐ray radiation for a few seconds [18]. Each of these methods can be used to estimate BA, but they may not be available everywhere. Although ionizing radiation can be harmful, the dose received during wrist and hand radiography is very low and comparable to everyday environmental exposure [37, 38]. Also, the cost of performing some of these methods is high. With these descriptions, radiography can be the best option for estimating BA (Figure 2). AI – involving particularly DL algorithms – has been adopted with enthusiasm for radiographs (X‐rays), especially for automating wrist and hand image analysis [6, 30]. With MRI, AI models can assist with delineation of bone structures and detect features of skeletal maturity with good accuracy, although their use is more limited due to a lack of standardised datasets [33]. In ultrasound, AI may assist with interpretation of echo patterns in the bone and improving image quality, but AI utilisation in this field is also relatively new [35].

#### 2.1.3 Organs

**FIGURE 3:**
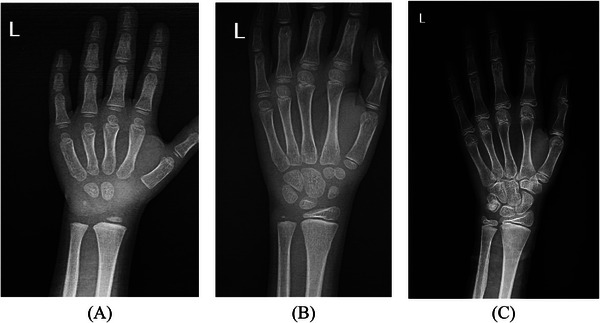
Images of the left wrist at different ages. Images of the left wrist of three children who have been referred to the radiology clinic to determine the BA. Image A is for a 4‐year‐old boy, image B is for an 8‐year‐old boy, and image C is for a 12‐year‐old boy. As you can see, the growth changes in the wrist area are quite evident. Changes in the growth of small bones in the wrist area as well as the joint surfaces of the fingers are among the things that are effective in determining BA. Radiologists can estimate BA based on these changes and with the help of existing atlases.

Different areas are usually used to determine BA, such as knees [32, 39], cervical [40, 41, 42], teeth [43, 44], ribs [36], elbows [9], and ankles [45], but wrists and hands are the most used in assessing BA. But due to the existence of BAA atlases, wrist and hand are more convenient and popular. To determine the BA, the method of using reference radiographs available in the atlas is usually used, which is the simplest and most accessible method to determine the BA and is usually suitable for determining the BA of both adults and children; however, for children, there will be a certain degree of mismatch. It shows the reference with radiography and includes the following two methods: the TW method, which is based on the development of 20 specific areas in the clear bones of the wrist and hand to determine the BA of different age groups, including children, in which a numerical score is defined for each stage of the development of each bone, and then the relevant doctor BA is obtained from the sum of these scores [46]. It should be noted that this method is more complicated and time‐consuming, but it is more accurate and repeatable than the GP method, where unique images are prepared according to age and gender standards. In these images, the analyses of the size, morphology, shape, and density of ossification centres in radiographs of children's hands are displayed [47]. Figure 3 depicts the radiographic view of the left wrist across various developmental phases often used in BAA according to the GP and TW methods.

#### 2.1.4 Datasets and Gold Standard

In evaluating BA, various data can be used for machine training. These data may be extracted from imaging clinics and hospitals. Also, some experts and researchers use prepared sources and data to design and implement AI algorithms. RSNA is one of the resources that many researchers use for automatic and intelligent assessment of BA. This resource was established by the RSNA for research activities and implementation of models based on AI. This collection includes 14,236 wrist and hand radiographs, which have been collected as the procedure for training, validation, and testing, which contains 12,611 training sets, 1425 validation sets, and 200 test sets [48]. Also, some websites are used as a source of data, such as Image Processing Information Lab.org [49]. In manual methods to assess BA, a radiologist can estimate BA based on experience and with the help of different atlases. In the methods proposed by researchers based on AI, one or more radiologists should report the medical images, and these images will be considered as a reference for training the machine. Therefore, the evaluation criterion of a model based on AI is based on the comparison of the results with the report of the radiologist [50].

### 2.2 The Power of Deep Learning in Medical Imaging

In the dynamic technological landscape, AI has emerged as one of the fastest growing and most influential innovations and offers a wide variety of applications in medicine and health care. In imaging methods, including radiological imaging, ultrasound, and MRI, AI allows more accurate and efficient diagnoses of diseases [51, 52, 53]. For example, specific AI algorithms allow for the detection and localisation of breast cancer lesions on the mammogram [54]. Echocardiography‐specific AI algorithms allow the detection of cardiac abnormalities [55]. ML, a specialised subset of AI, allows systems to learn and improve their performance from data, without the need for explicit programming. The typical workflow of ML methods includes data preprocessing, feature extraction, modelling, evaluation, and optimisation. DL, an advanced version of ML, uses hierarchical neural networks to automatically extract complex features from the raw data, in particular from medical images. The use of DL methods will reduce to a large extent the need for manual enhancements and lead to more rapid training and inference times using GPU‐based processing, allowing major improvements in speed and accuracy of diagnoses and throughput in clinical workflows [17]. In the DL architectures, ANNs provide the base framework in terms of concept. Among the most commonly used architectures, two of the most relevant types of DL are CNNs and recurrent neural networks (RNNs). CNNs have been used for BA estimation in the form of well‐known architectures, including LeNet, AlexNet, VGG, GoogLeNet, and ResNet, which allow spatial hierarchies to automatically learn from medical images with demonstrated improvements in terms of accuracy and robustness over traditional ML models [30, 56]. RNNs may have importance in that they allow for retention of temporal data and can exploit such processing ability when applied to medical images which have a temporal component [57].

### 2.3 Sensitivity Analysis

In this study, 50 articles related to the evaluation of various BA estimation methods using imaging technologies and DL were reviewed. Based on a qualitative assessment using the QUADAS‐2 tool, the findings showed that 66.0% of the studies had a low risk of bias in the “patient selection” domain, while 34.0% had a moderate risk and only 0% had a high risk. Regarding the applicability of patient selection, more than half of the studies (70%) were classified as having a moderate risk. For the “index test” domain, the majority of studies (86.0%) demonstrated a low risk of bias, with only 14.0% reporting moderate risk. In terms of applicability concerns for the index test, approximately 70% of the studies were rated as moderate risk. In the, “reference standard” domain, 88.0% of the studies had a low risk of bias, and 68% were assessed to have moderate applicability concerns. In the “flow and timing” domain, 40% of studies were rated as low risk and 60% as moderate risk, indicating a balanced distribution. These results indicate that, overall, the methodological quality of the studies was acceptable. However, the presence of moderate risk in some domains – particularly regarding the applicability of patient selection and the index test – highlights the need for greater rigour and attention in the design and conducting of future studies to enhance the validity and generalisability of the findings. The full item‐by‐item QUADAS 2 evaluation of all 50 studies is presented in Appendix Table 1. For conciseness, Table 1 summarises the frequency distribution (low, moderate, and high) of bias and applicability concerns across all QUADAS 2 domains.

### 2.4 Overview of Included Articles

#### 2.4.1 Aims of the Articles Extracted in This Study

The various aims of the articles are summarised in Table 2. In the upcoming collection, you can understand the importance of that topic based on the amount of data in the table in each topic. In this collection of articles, the main aim is related to creating algorithms and new models for automatic assessment. As you can see, MRI is the second imaging modality that has been used to evaluate BA in this series, and the purpose of the articles is to detect BA with the help of MRI. Determining BA with the help of ultrasound and CT scans and evaluating BA using software have been among the next important aims of these studies.

**TABLE 2:** Aims of the articles extracted in this study.

Aim	No. of study	Study
Deep automated skeletal BAA	20	[42, 43, 56, 58, 59, 60, 61, 62, 63, 64, 65, 66, 67, 68, 69, 70, 71, 72, 73, 74, 75, 76]
Determining chronological age using teeth	1	[65]
Unsupervised DL model	1	[77]
Validation and bias of DL models	1	[78]
Selecting the best approach for BAA	1	[79]
Ranking learning for BAA	1	[80]
Emerging research of DL models for BAA	1	[81]
Present the state‐of‐the‐art evidence, trends, and gaps in BAA with AI	1	[82]
Evaluate the accuracy and efficiency of a new automatic software system for BAA	2	[49, 83]
Investigate improvement in performance for automatic BA estimation	1	[84]
A review of the history of BAA with AI	1	[85]
Investigation of an automatic BA assessment method for Chinese children	1	[86]
Powerful graphical user interface (GUI) for image retrieval in medical applications (IRMA)	1	[87]
Automatic BAA using MRI	6	[31, 32, 33, 34, 88, 89]
Estimating human age using bone CT images	2	[36, 90]
Developing an AI‐based BAA model for Han and Tibetan children	1	[91]
Determining the best convolutional neural network model derived from state‐of‐the‐art architectures	1	[44]
Taxonomy of automated BAA approaches and discusses the challenges	1	[92]
Estimation of BA using ultrasound	2	[8, 35]
Estimation of BA using cervical vertebrae	1	[41]
A semi‐automated method for BAA using cervical vertebral maturation	1	[40]
Evaluation the performance of a deep neural network model in assessing rapidly advancing BA during puberty using elbow radiographs	1	[9]
Age estimation using ankle radiographic examination in a contemporary Thai population	1	[45]

In Table 2, the studies reviewed employed different methods for BAA based on the use of AI and imaging techniques with radiography, ultrasound, MRI, and CT. Most of the new studies [58, 74, 75, 76, 84] reported DL‐based automated skeletal BAA using wrist or hand X‐rays with an MAE usually below 0.6 years. CNN and variants (faster R‐CNN, DenseNet, and VGG) were widely used for region detection and feature extraction, with better performance than traditional approaches. Some studies also pursued newer avenues with promise for BAA, i.e., BAA with ultrasound [8, 35], which is promising, although less accurate than radiographic techniques, and cervical vertebra and elbow‐based skeletal models [9, 40], which represent alternatives which can be used for orthodontic and puberty‐related age estimation. The accuracy and robustness of results have improved with the use of ensemble methods [84] and ranking‐learning frameworks [80] when the multi‐model fusion and the monotonic loss constraints were combined. Some population‐specific adaptations were also reported, such as EVG‐BANet for Han and Tibetan children [91] and BX‐China05 for the setting up of a Chinese reference for BA [93]. The bias studies [78] observed that different demographics may result in diverse methodological outcomes, thus stressing the importance of diversity in datasets. The studies, overall, thus provide confirmation that there is a rapidly increasing impact on BAA from the use of DL, with challenges remaining especially in terms of generalisability, clinical applicability, and the integration of multimodal techniques. Following on from these observations, the articles reviewed in Table 2 provide further details regarding the main areas of research that are driving this area of research. Most of the cited works are focused on developing and verifying automatic algorithms for skeletal BAA using DL, and more specifically CNN‐based architectures, to improve diagnostic accuracy and performance [42, 43, 44, 58, 60, 67, 68, 69, 70, 71, 72, 73, 74, 75, 76, 77, 78, 79, 80, 81, 82, 83, 84]. X‐ray radiography, by far the most commonly utilised mode of imaging, is still predominantly based on left‐hand radiographs used for the estimation of skeletal maturity [40, 41, 45, 49, 60], with MRI being evaluated in a small number of studies as a complementary approach [31, 32, 33, 34, 95, 96]. There is less literature evaluation of ultrasound and CT modalities being employed; ultrasound is still not as reliable as conventional radiography can be [8, 35], while CT has been utilised in respect of certain specialised bones, e.g., ribs and ankles, as part of alternative approaches to age estimation [36, 97]. A number of studies have also pioneered methodologic advances and explored new paths for BAA. Some algorithms, employing circle of interest analysis on X‐rays of the hands, have produced a high degree of accuracy because different bones are weighted [84]. Some semi‐automatic methods utilising cervical vertebrae maturation on lateral cephalograms have produced strong correlation with clinical assessments by the expert examiners and have been valuable in orthodontic assessments [40, 41]. R‐CNN DL algorithms were able to detect ossification centres with increased accuracy (MAE approx. 0.5 years) [60, 91]. Furthermore, ensemble procedures which utilised different DL networks had lower rates of errors, while ranking‐learning structures provided robustness by producing monotonic loss functions [80, 84]. In recent times, several review‐related works have dealt with documenting the continuum of historic progressions, current state, and existing gaps regarding the application of DL in BAA [89, 90, 92]. Other studies have discussed the maturation of cognitive software tools and graphical user interfaces for easier retrieval and analysis of medical images to promote accessibility and workflow in the clinical environment [49, 61, 94]. Overall, these results emphasise the evolving and dynamic development of AI‐driven BA assessment and continued improvement towards accuracy, efficiency, and clinical result integration.

#### 2.4.2 Table of Information and Evaluation of Articles

In Table 3, useful and valuable information has been extracted from various articles. This information helps to create an accurate understanding of the work done in BA assessments. Race, datasets, evaluation criteria, implementation model, imaging method, and testing area are some of the things that are collected in this table. Also, in the following, reports are prepared based on Table 3. The selected articles in Table 3 have been screened based on their keywords and topics related to AI models and methodologies, and hence are focused yet comprehensive in the field.

**TABLE 3:** Table of information and evaluation of articles. This table contains complete information, such as data set, model, validation, and number of tests and training, extracted from the articles, which are expressed separately.

Dataset	Age range (years)	Train (No.)	Test (No.)	External validation	Modality	Race	Model	Organ	Evaluation criteria	Evaluation results	Reference
Digital hand atlas (DHA) [94]	0–18	1369	—	Two radiologists	Radiography	Asian, Black, Caucasian Hispanic	Region‐based CNN	Hand & wrist	MAE	The MAE between the radiologist's report and the proposed method is 0.48–0.51 years	[58]
House dataset and RSNA	0–18	240	—	A radiologist & RSAN	Radiography	—	Regression networks	Hand & wrist	MAD	The mean absolute difference (MAD) between radiologist reports and the proposed model is 4.56 months	[59]
House dataset	4–18	900	—	Med‐BA software	Radiography	Korean	Deeplabv3 Resnet‐v2	Cervical	MAE RMSE	The MAE and RMSE between the results of the proposed model and the Med‐BA software are 0.30 and 0.39 years	[42]
RSNA	2–18	960		—	Radiography	—	BA‐CCAE	Hand & wrist	MAE	The accuracy of the BA‐CCAE model compared to RSNA reports is 76.15%	[77]
RSNA	0–18	12,611	200	—	Radiography	—	Xception	Hand & wrist	MAE	The mean absolute error (MAE) between the Xception model and RSNA validation results is 8.17 months	[60]
RSNA	0–19	12,811	200	—	Radiography	—	Regression network	Hand & wrist	MAE	The differences between the proposed method and the RSNA validation results is 8.2 months	[61]
RSNA	0–18	8829	1891	—	Radiography	—	DCNN	Hand & wrist	Accuracy and F‐score	The accuracy between the DCNN model and the external observer (RSNA) is 97%	[79]
RSNA	0–18	80	20	—	Radiography	—	CNN network VGG‐U‐Net and GAN	Hand & wrist	MAE	The MAE between the age estimation of the implemented model and the RSNA validations is 6.05 months	[80]
RSNA	1–18	14,236	—	—	Radiography	—	Xception network	Hand & wrist	MAE MSE	The MAE between the results of the implemented model and the RSNA data is 7.6 months MSE: 108.8 months (The performance of the proposed model compared to other implemented models)	[62]
House dataset	3–17	200	—	Two radiologists	Radiography	—	Automatic software	Hand & wrist	Concordance rate and correlation coefficient	The concordance rate is 69.5% and the correlation coefficient *r* = 0.992 (comparison of software performance compared to BA reported by radiologists)	[83]
House dataset	0–6	345	87	—	Radiography	—	RCNN	wrist	K‐fold cross‐validation approach	Average discrepancy of 2.75 months between clinical and automatic BA evaluations and 90.15% accuracy	[63]
House dataset	—	15,129	214	Two radiologists	Radiography	—	TDL‐BAAM & GPDL BAAM	Hand & wrist	MAE	The MAE of the TDL‐BAAM model was 11.1 months and 12.9 months for GPDL‐BAAM. (The difference between radiologists' reports and models' predictions(	[56]
House dataset	5–18	5820	1248	Reports recorded by the radiologists in the clinic	Radiography	—	DCNN transfer learning	Hand & wrist	Average accuracy	Average BAA accuracy of 92.29% year (accuracy of model results compared to radiologist reports(	[64]
RSNA‐DHA	0–18	80% RSNA dataset & DHA	20% RSNA dataset & DHA	—	Radiography	—	DenseNet121, InceptionV3, and InceptionResNetV2	Hand & wrist	MAE	The MAE between model predictions and digital atlas data is 6.97 months. This is better compared to standard DL models, which are 9.41 months	[65]
House dataset	4–15	619	—	—	Radiography	Polish	DNN	Dental	MAE RMSE Correlation coefficient *R* ^2^	The MAE error of the produced models, depending on the learning set used, was between 2.34 and 4.61 months, while the RMSE error was between 5.58 and 7.49 months. The correlation coefficient *R* ^2^ ranged from 0.92 to 0.96	[43]
RSNA	4–18	400	200	—	Radiography	—	CaffeNet	Hand & wrist	MAD	Compared to the results of RSNA, the proposed artificial neural network can have an average measured absolute difference of 18.9 months and a correlation coefficient of 0.79	[67]
House dataset	—	745	240	Two radiologists	Radiography	Chinese	(ResNet)	Hand	MAD RMSE	The RMSE of AI was 0.76 years, and the MAD was 0.58 years (95% confidence interval, 0.55–0.62 years)	[86]
DHA	3–18	300	—	Two radiologists	Radiography	Asian	Age estimation software	Hand	Inter‐class correlation	The results obtained from the software and the radiologists showed correlation (ICC = 0.990 in male subjects and ICC = 0.986 in female subjects)	[95]
House dataset	0–18	2640	660	Two radiologists	Radiography	Korean	R‐CNNs VGGNet‐BA CNN	Hand & wrist	MAE RMSE	The MAE between the proposed method and the reports of radiologists is 0.46 years. Also, the RMSE is equal to 0.62 years	[68]
House dataset	14–21	402	—	—	MRI	Swedish	CAFFE	Knee	MAE	The proposed method was able to assess the age of male subjects in the range of 14–20.5 years, with an MAE of 0.793 years, and of female subjects in the range of 14–19.5 years, with an MAE of 0.988 years	[88]
RSNA	0–18	300	—	—	Radiography	—	VGG‐16; VGG‐19 ResNet‐50 Inception‐ResNet‐V2 Xception	Hand	MAE Concordance correlation coefficient (CCC)	The MAE for the best running model compared to RSNA data is 8.59 months, and the CCC value is 0.94%	[69]
RSNA	0–18	352	—	—	Radiography	—	AlexNet; ResNet	Hand	Accuracy Specificity Precision Recall F‐measure	The results of the AlexNet method compared to RSNA data were as follows: accuracy: 99.3; specificity: 99.9; precision: 99.47; recall: 99.57; F‐measure: 99.59. The results of the ResNet method compared to RSNA data were as follows: accuracy: 99.2; specificity: 99.86; precision: 99.61; recall: 98.22; F‐measure: 98.87	[70]
KCRD data set (House dataset) and RSNA	0–18	5305	—	—	Radiography	Turkish	IncepitonV3 MobileNet EfficientNet	Hand & wrist	MAE RMSE	Among the three implemented models, the InceptionNet method obtained the best results. With the House dataset, MAE: 4.3 months, RMSE: 5.76 months With RSNA, MAE: 4.3 months, RMSE: 7.4 months	[72]
RSNA	0–16	8800	3811	—	Radiography	—	RCNN ELM	Hand & wrist	MAE RMSE	The results of the proposed model compared to RSNA data are as follows: MAE: 6.07 months and RMSE: 11.48 months	[73]
House dataset	0–17	830	237	—	Radiography	Hong Kong	Faster RCNN; FCN; DenseNet	Radius & ulna	Recall, precision, F1‐score	The proposed method was carried out in two ways. One as an individual and the other as a group, and the results were obtained as follows. In the individual method, recall: 79.65%, precision: 74.44%, F1‐score: 76.96% Results of the group method: recall: 83.33%, precision: 81.08%, F1‐score: 82.19%	[74]
House dataset	8–87	569	244	—	CT	—	VGGNet; GoogLeNet; ResNet	Whole body	MAE	The results of the proposed method based on gender and compared to the radiologist's report are as follows: female: 4.351 months, male: 6.969 months	[90]
RSNA	0–18	825	351	—	Radiography	Tibetan Han	EVG‐BANet	Hand & wrist	MAD	MAD and accuracy as performance indicators in the implemented model compared to RSNA data were as follows: 0.62 years, *p* < 0.001	[91]
House dataset	0–6	195	—	—	Radiography	Asian African‐American Caucasian Hispanic	Support vector machine (SVM), Naive Bayes, k‐nearest neighbourhood, and C4.5 algorithms	Wrist	Accuracy	In this article, four methods were implemented to predict bone age. The SVM method obtained the best result with 96.41. (prediction accuracy compared to the report of radiologists)	[96]
House dataset	19–90	4035	—	—	Radiography	Croatia	VGG16	Dental	Coefficient of determination (𝑅^2^). MAE	The results of the trained model in the panoramic images that include all the teeth and the jaw area are as follows: MAE: 4.06 years, and 𝑅^2^: 0.8405 Model results when the evaluated area is only one tooth or a limited area: MAE: 4.65 years, *R* ^2^: 0.78	[44]
RSNA	0–19	12,611	1136	—	Radiography	—	VGG16	Hand & wrist	MAD	The MAD between the VGG16 model results and the RSNA data is 6.8 months	[75]
House dataset	13–23	240		Two radiologists	MRI	Caucasian	DCNN	Hand	Mean error	The mean error of BA estimated by the proposed model and the reports of two radiologists is 0.36 ± 0.30 years	[34]
Simulation	6–13	90	45	Two radiologists	Radiography		Computer vision algorithm	Hand & wrist	MSE	Measuring the mean squared difference between predicted values and radiologists' reports is 0.0865	[76]
House dataset	13–20	132	—	—	MRI	—	Random regression forests	Hand	Absolute deviation	0.82 ± 0.56 years of absolute deviation from chronological age	[89]
House dataset	11–16	252	—	Two radiologists	MRI	—	Linear regression analysis	Hand & wrist	Pearson's correlation coefficient (*R* ^2^)	Correlation between chronological age and model implemented with Pearson's correlation evaluation criteria: *R* ^2^ = 0.9	[33]
House dataset	5–15	37	—	A radiologist	Ultrasound	Japanese	Linear regression	Wrist	IME	The inter‐measure error of the BA determination method using ultrasound and radiography is +0.36–0.54 and the correlation is *R* = 0.89	[35]

In Table 3, a few studies used ultrasound; the authors [35] analysed the application of the Sunlight BonAge (SBA) ultrasound device for BA determination in 37 children. The SBA results were compared to the reference radiographic methods – TW2 (RUS) and CASMAS. The comparison with the TW2 and CASMAS reference methods showed correlation coefficients of 0.89 and 0.85, respectively. The authors also reported low inter‐measurer (0.54 ± 0.36 years) and inter‐measurement errors (0.51 ± 0.42 years), indicating good reproducibility of the ultrasound‐based method. The paper [89] introduces a regression‐based approach for estimating radiological BA using 3D images of the hand from MRI data. By merging multiple bones into a single composite model, the approach evolves from ageing being a singular event to being a global phenomenon, reflecting the way ageing occurs. The proposed model produces a mean absolute deviation (MAD) of 0.82 ± 0.56 years. The authors [34] focus on biological age estimation in the second article using deep CNNs. This is inspired by the way doctors typically perform visual estimations based on established staging criteria. This approach leverages hand MRI volumes to automate the process. Their method significantly outperforms existing ones, with an average deviation of only 0.36 ± 0.30 years between the predicted and actual biological age. The results align well with expert radiologists' manual estimates, suggesting that this model is reliable for both clinical diagnostics and legal applications, such as confirming endocrinological disorders or aiding paediatric surgical procedures. A study by Wang et al. [74] explores the possibility of utilising whole‐body CT scan images of children to develop a model for estimating BA. This provides a departure from the conventional model of estimating BA on the basis of hand X‐ray images. Using a modified version of VGGNet on 813 whole‐body CT images, the researchers demonstrated that the major upper body skeletal features are useful for estimating BA. The model provided some indication of gender differences in accuracy, and, importantly, provided some evidence that the architecture of the model could be simplified to improve results when applied to small datasets. The article [44] implemented DL for forensic dental age estimation using panoramic dental X‐rays to arrive at a record‐low error (2.95 years) for adults/seniors where pre‐trained CNNs and related ablation techniques were incorporated. An article by Zaborowicz et al. [43] implemented deep neural networks for dental age estimation and also reported very high accuracy (MAE: 2.34–4.61 months; *R* ^2^: 0.92–0.96) showcasing the gained advantages of AI over manual approaches. Dallora et al. [88] developed an MRI‐based BAA model for youth (ages 14–21) by implementing a two‐stage CNN and reported MAEs below 1 year (MAE = 0.793 years for males, 0.988 years for females) representing a very accurate and reliable option that does not include radiation methods like X‐ray. The study [33] examined MRI's feasibility for BAA by retrospectively evaluating hand and wrist images of 179 teenagers, 11–16 years of age. A low‐field open magnet at 0.2 Tesla was used to acquire T1‐weighted images, which were then evaluated by two blinded evaluators for a variety of bone maturity indicators, including that of cartilage advancement and ossification. The results showed that there was a strong correlation, *R* ^2^ = 0.9, between MRI‐based BA and chronological age, with good interobserver reliability. This means that MRI‐based methods could offer a feasible alternative in the development of age estimation atlases without radiation exposure.

A study by Seo et al. [42] utilised lateral cephalograms to develop a DL model to estimate BA using cervical vertebrae, resulting in a low MAE of 0.30 years for the model which utilised DeepLabv3+ for segmentation and InceptionResNet‐v2 for regression. The authors concluded that the model framing the problem using lateral cephalometric X‐rays has potential as a less harmful alternative to hand‐wrist X‐rays, as it is associated with lower radiation exposure in the hands when viewing longitudinal images of the same participant via the lateral skull X‐ray. A study by Koitka et al. [59] described a two‐step neural network modelled on the TW method called CWBMD‐2. The network was able to achieve a mean error of 4.56 months on sequential scans from the RSNA dataset and maintained interpretability by highlighting the areas of ossification with respect to the age of the participant, making it suitable for clinical decisions for practitioners in healthcare. Son et al. [68] developed and presented a full DL pipeline to estimate BA based on the TW‐3 model using a unique dataset comprising 3300 X‐rays. The final system achieved a top‐1 accuracy of 79.6% and an MAE of 0.46 years. The system outperformed currently available commercial systems using GP‐based solutions, illustrating its performance and clinical utility. Lee et al. [64] utilized an automated BAA framework based on U‐Net segmentation for active learning and to minimize manual annotations. The framework integrated segmentation, image standardisation, and a fine‐tuned CNN. The accuracy was very high for females: 90.39% under 1 year and 98.11% under 2 years; for males, the accuracy was 94.18% and 99.00%, respectively. The attention maps highlighted that the learning from the model aligned with expert evaluation of BAA. Kim et al. [83] utilised 3300 X‐rays and modified CNNs to evaluate an automated system for BAA based on the TW‐3 method. The model could localise 13 bone regions and reported top‐1 and top‐2 accuracy of 79.6% and 97.2%, respectively, and an MAE of 0.46 years. The accuracy of the model was better than commercially available automated systems based on GP. Reference [63] took a different approach, but using a fully automated, CNN‐based pipeline to perform BAA, along with radiograph standardisation and segmentation. The top‐1 accuracy of the model was 57.32 and 61.40 for females and males, respectively. More than 90% of the attention maps were aligned with predictions that fell within one year of the ground‐truth bone age. The attention maps were also useful and demonstrated interpretability, which has potential in a clinical system decision support. In the study by Lee and Kim [67], the development and validation of an automatic software system for BAA using DL were discussed. A concordance rate of 69.5% with radiologist‐determined reference BA was demonstrated by the system, which was based on the GP method. The integration of the software with human reviewers resulted in improved concordance rates and a reduction in reading times by up to 40%. It was concluded that the system increased both accuracy and efficiency in clinical practice without compromising diagnostic reliability. In a study by Mualla et al. [70], a method that leans on DL automation for figuring out BA was put forward. This method taps into a special kind of neural network focused on regression to study the wrist bones in X‐ray images of little kids. In the studies conducted, it was found that there was a typical gap of about 2.75 months when comparing doctors’ findings with those from computer‐based checks. These automated methods were right about 90.15% of the time if you look within a six‐month period of the actual facts. Through the approach used, it became clear that there was a strong consistency with the traditional hand‐checked evaluations, showcasing its dependability. Haghnegahdar et al. [95] introduced a novel method for estimating age based on hand X‐rays, framing the issue as a regression task through the application of DL techniques. The methodology involved preprocessing the radiographs to reduce variations not related to age. For instructional purposes, a straightforward CNN was utilised. Validation results indicated a MAD of 18.9 months and a concordance correlation coefficient of 0.78. This approach is recognised for its ability to enhance the efficiency of age estimations in clinical settings by removing the necessity for manual reference to atlases. The DL model presented in [50] utilised 15,000 paediatric hand radiographs to achieve an MAE of 11.1 months. It outperformed GP‐based practices and expert radiologist diagnoses. Although found to be substantially concordant with chronological age, there was evidence of the model overestimating age for younger children. Panayides et al. [51] developed a decision support system, using DenseNet121 and InceptionV3‐based networks, to estimate BA for segmented regions of the hand. Advances made through the development of a new region‐based feature‐connected layer improved feature representation, resulting in an MAE of 6.97 months that was, at a minimum, superior to traditional approaches. In a paper by Ting et al. [52], an AI‐based BAA system was evaluated with 8000 Chinese paediatric radiographs, with determined accuracies of 84.6% within one year of the ground truth. The model was found to be consistent with expert performance overall, and the authors acknowledged limitations with bone deformities and different maturation rates. Lee et al. [64] utilised an automated BAA framework U‐Net segmentation for active learning and to minimise manual annotations. The framework combined feature extraction from deep CNNs and ensemble regression analysis to provide an average error of about 7 months, which was better than the state‐of‐the‐art techniques. In [66], an automatic system for BAA, the so‐called Attention‐Xception network (AXNet), was developed to overcome the weaknesses of the traditional methods. AXNet introduced image normalisation and a regression module by taking advantage of spatial‐attention mechanisms that highlight important features. Experimental results manifested that the AXNet had the smallest MAE, which made it suitable for less than one year of error margin in clinical practices. Lee and Kim [67] discussed research on a comparative study regarding an automated system that uses CNNs to assess BA. This model, which was trained on the RSNA dataset, was used to predict BA from left‐hand X‐rays. They employed various evaluation metrics, including but not limited to accuracy, recall, precision, and F‐score. The accuracies for this proposed model achieved a test accuracy of 97%, and hence proved its potential for assisting health professionals in clinical use. In the investigation [70], an image‐based model for assessing BA was proposed based on the Xception architecture. The model emphasised transfer learning to extract features related to bone maturity. It then used more than 12,000 hand radiographs from the Kaggle RSNA BA dataset. Overall performance yielded an MAE of 8.175 months, meeting the goal of developing a system that estimates the BA for male and female patients within one year of actual BA.

## 3 Discussion and Conclusion

The useful information and results of each article were extracted. This information allows the data to be described quantitatively and qualitatively. The data and results of each article can be seen in Table 3. The field of diagnostic imaging has undergone incredible transformations with the introduction of automated and accurate approaches to clinical evaluation due to advances in AI technology. Such AI‐based systems in the field of BAA illustrate the potential for a remarkable reduction in diagnostic time as well as improved accuracy of the examinations performed. The studies reviewed in this paper showed that the most common sources of data were in‐house (datasets collected from hospitals and clinics) and public series such as the RSNA dataset [8, 35, 46]. In‐house databases have certain important advantages, such that the results can be analysed in terms of race, geography, and other environmental influences which may have an effect on the skeletal maturation of any population. This is particularly important to consider, as it is well known that population groups exhibit differences in the rate of growth of bone. For example, the rate of growth of African children is greater than that of Chinese children, and this is important when analysing models suitable for the populations. On the other hand, the RSNA dataset contains a large dataset of over 14,236 hand radiographs which have been annotated by paediatric radiologists and which form a standardised, extensive, and well‐validated yardstick [66]. The use of such extensive datasets enables models which incorporate diversity and reproducibility of results as well as another important element of efficient training and validation of AI‐based models. Across the literature, it appears that the most widely used method of assessing the quality of BAA models is the MAE value [17, 30, 58, 78]. A decreased MAE value is indicative of greater accuracy of the model and of a closer relationship to the opinion of expert radiologists. In the majority of studies which apply deep‐learning methods, the MAE values reported have been less than 0.6 years, indicative of the ever‐increasing ability of AI models to produce clinically acceptable results [74, 75, 76]. The fundamental goal of such intelligent systems is to provide reliable support to radiologists and endocrinologists and consequently reduce human workload and interobserver variation. A reliable model must therefore have not only low error but also a short computing time in order to provide near real‐time prediction and thus to assist in the efficiency of busy clinical workflows [9, 40]. The methods of ML which have most successfully achieved the task of BA estimation in literature remain the CNNs. The multi‐layer structure of such algorithms permits automatic feature extraction, starting from the raw X‐rays, and this removes the need for manual pre‐processing of the data. The region‐based CNN (R‐CNN) framework has proved to be especially successful for the task of anatomical label image classification since it allows the automated identification of anatomical regions of interest such as the wrist and hand bones and the study of their patterns of growth and has been subjected to a reliable review of literature [30]. Owing to the hierarchical principle of learning of features, the R‐CNN models are able to detect small dissimilarities between the various skeletal systems and are less sensitive to different datasets and overfitting. The efficacy and robust nature of these systems are the reason why it is found that any of the CNN‐based models outperform such classical algorithms, and they remain the principle driving force in the research concerning automatic BAA methods [58, 75].

Apart from the conventional CNN, the literature has been prompted to create new models to improve consistency yet further and enlarge the concept of the generalisability of the models. The ensemble methods deploy the outputs of several classifiers together for the task of creating predictive consistency. In a different direction, ranking‐learning‐based methods have arrived, which model the nonlinear and progressive nature of bone maturation [80, 84]. Some studies have been prompted to integrate hybrid techniques which consist of combining the conventional form of interpretation of the radiograph with the CNN‐based methods of feature extraction to improve predictive precision or clinical‐based interpretational and communication approaches [9, 91].

**FIGURE 4:**
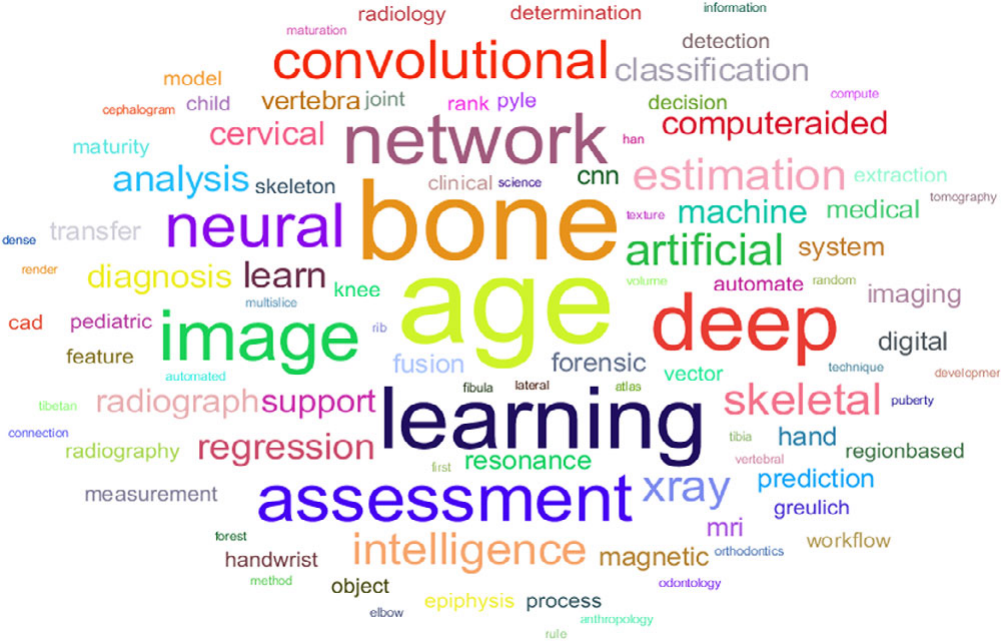
Visual representation of keywords in reviewed articles.

Alternative approaches have been deployed for populations, such as specific models developed for either Han or Tibetan children or the new BI‐China05 reference scale [91, 94]. These attempts highlight the necessity for demographic adaptation and localised datasets collectively, and the reduction of biases and equal performance of AI across populations. Despite the significant development, many issues still present themselves. One of these is the commonly occurring question of the over‐ or under‐scoring of the BA in certain sub‐groups, which is particularly common in cases of delayed or accelerated maturation [8, 35]. A second issue is in obtaining enough generalisability for the models employed when those from one dataset are applied to different demographic populations. Allaying these disadvantages can be accomplished through the application of diversity to the training data and performing across multiple datasets and external validations. In addition, the interpretability and transparency of AI predictions support their acceptance in clinical practice. The radiologist must be able to understand the logical justification behind the output of the models employed, especially in cases where the prediction given is at variance from that of the expert opinion [51, 78]. The ethical and regulatory questions associated with the use of AI in medicine are another key and central facet of the various guises in which these vast computational tools can be used. As the size and complexity of the dataset increase, so too arise the various questions of patient confidentiality and security of the data [97]. Such international dictates as the General Data Protection Regulations (GDPR) in Europe and the Health Insurance Portability and Accountability Act (HIPAA) in the USA create very high standards of required compliance with reference to the anonymisation, methods of secure storage, and confined access protocols to be employed for the patient datasets [21, 98]. The clinical applicability of AI‐based BA models at this point in time relies not just on their technical performance but also on their practical usability. In order for radiologists and endocrinologists to embrace the use of these systems, they must also provide obvious benefits over existing systems, greater speed of reporting, reproducibility of results, and clarity in the decision‐making process [9, 30]. Studies have shown that physician diagnostic consistency can be improved by the assistance of AI, both with regard to diagnostic reasoning and reproducibility; this reduces the variability between observers and improves the overall accuracy of diagnostics [30]. However, in order to obtain total clinical acceptance, the models would have to be validated by diverse and well‐annotated datasets and also with standardised test protocols. This will require collaboration between software developers, radiologists, and medical agencies in order to transform these systems from laboratory prototypes to dependable clinical tools [48, 51]. In the future, there are many fruitful lines of research. The use of transformer‐based architectures is one way in which AI systems may be more powerful at capturing longer distance dependencies and contextual patterns present in radiographs and may outperform the existing systems based on CNNs [99]. The development of hybrid frameworks utilising traditional imaging techniques with modern DL types of techniques will be another avenue of research that could improve diagnostic robustness [17]. There is also a need for longitudinal studies designed to monitor bone development over a significant period of time in order to gain more knowledge about the various growth trajectories and to develop predictive models that relay the expected change over time of the structure of the skeleton [97, 99]. Finally, it will be necessary to widen the training datasets to involve populations of various racial backgrounds and geographic locations in order to increase the generalisability of any further systems developed [91, 94]. The ultimate goal of AI‐based BAA is to help radiologists and not to replace them. By automating repetitive measures, minimising variation and improving reporting efficiency, AI can serve as a useful adjunct in clinical decision‐making [17]. Figure 4 presents the most frequently occurring keywords in the reviewed articles, indicating that research is primarily focused on bone age assessment using deep learning approaches. The use of words such as image, convolutional, and neural network is a further indicator of the application of AI‐driven imaging and computer vision techniques to BAA in today's studies.

The future of AI in BAA is about transparent, ethical, and inclusive development, where the algorithms are explainable, validated across populations, and compliant with data protection standards [98]. This will ensure that these systems reach high levels of accuracy and the confidence of the patients and other health professionals. The continuous evolution of AI technologies and their judicious application in the field of radiology can revolutionise diagnostic efficiency, standardisation, and ultimately patient care [34, 97].

## 4 Summary of Findings

This systematic review demonstrated that CNN‐based models, particularly R‐CNN, show superior performance in estimating BA from radiographic images. The use of diverse datasets – including internal (house) data and publicly available datasets such as RSNA – significantly enhanced the accuracy and generalisability of the models. Evaluation metrics such as MAE were commonly used across studies, indicating a high level of agreement between predicted and actual BAs. In addition to accuracy, execution speed and processing time are critical factors for the clinical adoption of these models by radiologists and endocrinologists. Furthermore, incorporating racially and geographically diverse data, along with validation from experienced radiologists, has contributed to increasing the reliability and credibility of the outcomes.

## 5 Research Gaps and Future Directions

Despite the considerable advancements in this field, several important challenges remain. First, the lack of racially and environmentally diverse training data limits the generalisability of current models. Second, there is a notable scarcity of longitudinal studies capable of modelling bone development over time, which is essential for more accurate age predictions. Developing hybrid models that combine traditional imaging techniques with advanced DL algorithms can potentially improve model performance and reduce error rates. Moreover, ethical considerations such as patient privacy and model transparency must be carefully addressed to build trust among clinicians and support broader clinical implementation. Future research should aim to address these gaps in order to further enhance the clinical utility, robustness, and inclusiveness of AI‐based BAA systems. Another important issue that requires attention is publication bias. Publication bias occurs when studies reporting positive or significant results are more likely to be published than those with negative or null findings, which can lead to an overestimation of model effectiveness. To accurately assess the reliability and generalisability of DL models, evaluation methods such as funnel plots and sensitivity analyses are essential. These techniques help identify and quantify potential biases in the literature, ensuring more robust and unbiased conclusions. Future research on DL in medical imaging should incorporate these evaluation methods to improve transparency and trustworthiness of reported outcomes.

## Author Contributions

MB, MY, and FBM wrote the paper and participated in the revisions of it; MB and MY performed the literature search and data analysis.

## Funding

The authors have nothing to report.

## Conflicts of Interest

The authors declare no conflicts of interest.

## Data Availability

Not applicable.
